# User-Centered Design of a Mobile Health Intervention to Enhance Exacerbation-Related Self-Management in Patients With Chronic Obstructive Pulmonary Disease (Copilot): Mixed Methods Study

**DOI:** 10.2196/15449

**Published:** 2020-06-15

**Authors:** Yvonne J G Korpershoek, Sander Hermsen, Lisette Schoonhoven, Marieke J Schuurmans, Jaap C A Trappenburg

**Affiliations:** 1 Research Group Chronic Illnesses University of Applied Sciences Utrecht Utrecht Netherlands; 2 OnePlanet Research Center imec NL Wageningen Netherlands; 3 Julius Center for Health Sciences and Primary Care University Medical Center Utrecht Utrecht Netherlands; 4 Education Center UMC Utrecht Academy University Medical Center Utrecht Utrecht Netherlands

**Keywords:** mobile health, mHealth, user-centered design, behavior change, COPD, exacerbation, self-management, self-care, mobile phone

## Abstract

**Background:**

Adequate self-management skills are of great importance for patients with chronic obstructive pulmonary disease (COPD) to reduce the impact of COPD exacerbations. Using mobile health (mHealth) to support exacerbation-related self-management could be promising in engaging patients in their own health and changing health behaviors. However, there is limited knowledge on how to design mHealth interventions that are effective, meet the needs of end users, and are perceived as useful. By following an iterative user-centered design (UCD) process, an evidence-driven and usable mHealth intervention was developed to enhance exacerbation-related self-management in patients with COPD.

**Objective:**

This study aimed to describe in detail the full UCD and development process of an evidence-driven and usable mHealth intervention to enhance exacerbation-related self-management in patients with COPD.

**Methods:**

The UCD process consisted of four iterative phases: (1) background analysis and design conceptualization, (2) alpha usability testing, (3) iterative software development, and (4) field usability testing. Patients with COPD, health care providers, COPD experts, designers, software developers, and a behavioral scientist were involved throughout the design and development process. The intervention was developed using the behavior change wheel (BCW), a theoretically based approach for designing behavior change interventions, and logic modeling was used to map out the potential working mechanism of the intervention. Furthermore, the principles of design thinking were used for the creative design of the intervention. Qualitative and quantitative research methods were used throughout the design and development process.

**Results:**

The background analysis and design conceptualization phase resulted in final guiding principles for the intervention, a logic model to underpin the working mechanism of the intervention, and design requirements. Usability requirements were obtained from the usability testing phases. The iterative software development resulted in an evidence-driven and usable mHealth intervention—Copilot, a mobile app consisting of a symptom-monitoring module, and a personalized COPD action plan.

**Conclusions:**

By following a UCD process, an mHealth intervention was developed that meets the needs and preferences of patients with COPD, is likely to be used by patients with COPD, and has a high potential to be effective in reducing exacerbation impact. This extensive report of the intervention development process contributes to more transparency in the development of complex interventions in health care and can be used by researchers and designers as guidance for the development of future mHealth interventions.

## Introduction

### Background

Chronic obstructive pulmonary disease (COPD) is a highly prevalent chronic disease and is predicted to be the third leading cause of mortality worldwide in 2030 [1,2]. Exacerbations are important events in the course of COPD, as they accelerate the decline in lung function [3], negatively affect the quality of life [4,5], and lead to increased mortality and high socioeconomic costs [6,7]. An exacerbation is defined as *a sustained worsening of patients’* *respiratory symptoms, which are beyond normal day-to-day variability and may warrant medical treatment* [8]. The absence of an adequate imminent exacerbation marker requires a focus on supporting patients with COPD in developing self-management skills to reduce the impact of exacerbations [9]. Self-management is defined as *an individual’s ability to detect and manage symptoms, treatment, physical and psychosocial consequences, and lifestyle changes inherent in living with a chronic condition* [10].

Recent interventions focusing on exacerbation-related self-management (including the use of action plans) have shown positive outcomes on quality of life and hospital admissions [11,12]. However, there is still a substantial proportion of patients with COPD who barely benefit from these kinds of interventions [11-13]. This might be explained by the *one-size-fits-all* and static approach regarding design, intensity, and mode of delivery without a focus on individual exacerbation patterns and actions. Moreover, recent interventions have a strict focus on exacerbation detection and taking action and the use is suboptimal [11,14,15]. To further reduce the impact of exacerbations, more comprehensive, dynamic, and individualized strategies are needed to improve the full spectrum of exacerbation-related self-management behavior that meet patients’ needs, perceptions, and capabilities [12,16].

Mobile health (mHealth) is considered promising in engaging patients in their own health and changing health behaviors [17,18]. The rapidly evolving nature and increased uptake of mHealth are bound to influence the accessibility and the way self-management support will be provided in the future, also in patients with COPD [19-21]. Recent studies suggest that mHealth interventions focusing on COPD self-management lead to positive outcomes, although no firm conclusions could be drawn because of poor quality and heterogeneity among the studies [19,20]. Nonetheless, the use of mHealth creates opportunities to strongly individualize interventions and to provide more dynamic and intensive therapeutic stimuli that fit with real-time health status and individual exacerbation patterns. As a result, mHealth can reach patients at the right moment and can provide tailored support anytime and anywhere, which could stimulate the development of effective self-management skills and change health behaviors.

To date, there is limited knowledge on how to design mHealth interventions that are effective, meet the needs of intended end users, and are perceived as useful [17,22]. Designing mHealth interventions to change health behaviors is complex and needs theoretical grounding to increase the design’s efficacy. In current thinking about the development of behavior change interventions, the importance of theory is clear [23-26], but the way in which theory should be incorporated in the design process is not [24,27,28]. Furthermore, specific steps in the development of evidence- and theory-driven interventions that involve the end users are rarely described transparently in literature [22,29].

### Objectives

During a 4-year period, our research team has developed an evidence-driven and usable mHealth intervention to enhance exacerbation-related self-management in patients with COPD. By following an iterative user-centered design (UCD) process, several studies were performed to increase the likelihood of developing an mHealth intervention that is effective, fits with patients’ needs and preferences, and can be successfully implemented in routine COPD care. Some of these studies have recently been published [15,30,31]. This paper underpins the design and working mechanism of this COPD-specific mHealth intervention and offers a novel and potentially effective method to use evidence and theory to inform the design of mHealth interventions in general.

The aim of this paper was to describe in detail the full UCD and development process of an evidence-driven and usable mHealth intervention to enhance exacerbation-related self-management in patients with COPD, including the design, iterative software development, and usability testing.

## Methods

### User-Centered Design Process

**Figure 1:**
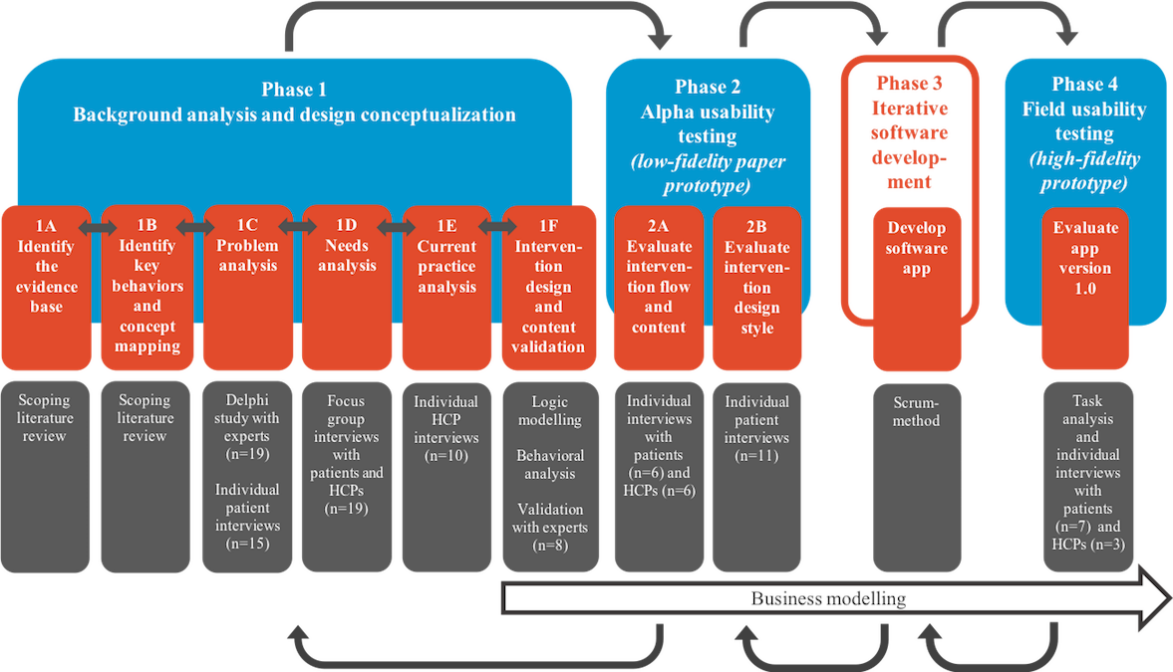
User-centered design for the development of the mobile health intervention. HCP: health care provider.

Guiding principles for the mHealth intervention were formulated by the research team at an early stage to provide a framework for making decisions during intervention development (Textbox 1) [32]. The guiding principles were based on recent evidence regarding COPD self-management and were progressively refined as the intervention development proceeded based on outcomes of specific development steps that we described in this paper. The mHealth intervention was developed by following a UCD process involving patients with COPD, health care providers (HCPs), COPD experts, designers, software developers, and a behavioral scientist. The UCD was based on the methodology as described by Johnston et al [33] consisting of four iterative phases: (1) background analysis and design conceptualization, (2) alpha usability testing, (3) iterative software development, and (4) field usability testing (Figure 1) [33]. Johnston et al [33] provide limited guidance on the specific steps needed to develop an effective mHealth intervention that meets patients’ needs and preferences and fits with current COPD care. Therefore, we extended the first phase of the UCD with subphases based on a comprehensive approach that combines elements of the Medical Research Council (MRC) framework development phase with elements of existing development models (Figure 1) [34]. The MRC framework is a well-known and often used framework for the development and evaluation of complex interventions in health care with a specific focus on developing theory- and evidence-driven interventions. The whole design and development process was carried out between 2015 and 2019. The methods of each phase are chronologically described in the following paragraphs. The results of each phase are detailed in the Results section.

**Guiding principles for a mobile health intervention to enhance exacerbation-related self-management in patients with chronic obstructive pulmonary disease.**

The mobile health intervention should:

- meet individual patient needs, perceptions, and preferences regarding exacerbation-related self-management;
- synchronize with current health status and anticipate on the heterogeneity of exacerbations in and between patients;
- focus on target behaviors in the full spectrum of exacerbation-related self-management;
- include a chronic obstructive pulmonary disease (COPD) action plan along with ongoing self-management support;
- focus on the continuous development of self-management skills and behavior change;
- stimulate proactive self-monitoring;
- be safe, literacy-sensitive, and patient-friendly;
- be feasible in current Dutch COPD care; and
- meet the conceptual definition of a COPD self-management intervention: *A COPD self-management intervention should be structured but personalized and often multicomponent, with goals of motivating, engaging and supporting the patients to positively adapt their health behavior(s) and develop skills to better manage their disease* [16].

#### Phase 1: Background Analysis and Design Conceptualization

The aim of the first phase was to identify the evidence base and to achieve a theoretical understanding of the underlying process of change for the intervention [35].

##### 1A: Identify the Evidence Base

In phase 1A, a scoping literature review was performed in Medical Literature Analysis and Retrieval System Online (MEDLINE) to explore current systematic reviews on interventions that focus on enhancing exacerbation-related self-management in patients with COPD, including mHealth interventions, and to identify potential effective intervention components (Figure 1; phase 1A). Literature review on interventions was an ongoing process during the whole intervention development process, to stay up to date on developments about (mHealth) interventions focusing on exacerbation-related self-management.

##### 1B: Identify Key Behaviors and Concept Mapping

In phase 1B, a scoping literature review was performed in MEDLINE to specify symptom fluctuation phases during the course of COPD and to identify relevant self-management behaviors that can reduce exacerbation impact (Figure 1; phase 1B). Two researchers (YK and JT) developed a conceptual model of patients’ fluctuations in symptoms during the course of COPD. Then, an initial set of relevant self-management behaviors was generated and added to the conceptual model. The methods of the scoping review and stepwise development of the conceptual model are published elsewhere [15].

##### 1C: Problem Analysis

A problem analysis was included to provide insight into the problems experienced by patients and identified by experts to determine the intervention targets and to set boundaries of the intervention (Figure 1; phase 1C). A two-round Delphi study with 19 international respiratory experts (medical doctors and key researchers in the field of COPD) was performed. In this study, insight into expert opinion was provided on the most relevant set of self-management behaviors that have the potential to maximally reduce the impact of exacerbations and is feasible to target and influence before, during, and after an exacerbation. The methodology is described in depth in the publication of this study [15]. Furthermore, a grounded theory study using individual in-depth interviews with patients with COPD (n=15) was performed [36]. In this study, patient perceptions, capabilities, and needs with regard to exacerbation-related self-management were explored to identify and explain the underlying process of exacerbation-related self-management behavior in patients with COPD. The methodology is described in depth elsewhere [30].

##### 1D: Needs Analysis

Patients' needs regarding exacerbation-related self-management were partially identified in phase 1C because these needs flowed naturally from the problems perceived by patients [37]. An additional needs analysis was performed to further investigate specific needs and explicit requests for care with regard to using mHealth for self-management (Figure 1; phase 1D) [37]. To develop an mHealth intervention with optimal usability and feasibility, a deep and early understanding of both patients’ and HCPs’ perspectives was considered to be important [38]. Therefore, a qualitative study using focus group interviews with both patients with COPD (n=13) and HCPs (n=6) was performed to (1) explore their willingness to use mHealth for self-management of exacerbations, (2) identify potential benefits and barriers of using mHealth, and (3) explore needs and preferences regarding the content of an mHealth intervention [39]. The methods of this step are further described in the paper of this study [31].

##### 1E: Current Practice Analysis

An analysis of COPD guidelines and current practice was performed to gain insight into current exacerbation-related self-management support and to explore the added value of the intended intervention compared with regular care (Figure 1; phase 1E). Individual semistructured interviews with HCPs (n=10) were performed to identify HCPs’ perspectives with regard to care provided and their role in providing self-management support. Purposive sampling was performed in primary and secondary care settings. The following topics were discussed: current interventions to support exacerbation-related self-management, HCP experiences with providing self-management support, perceptions toward HCPs’ roles and responsibilities, barriers in providing self-management support, and the potential to use mHealth for self-management support. All interviews were audiotaped, transcribed verbatim, and analyzed by open, axial, and selective coding [40].

##### 1F: Intervention Design

The aim of the intervention design phase (Figure 1; phase 1F) was twofold: (1) to map out the potential working mechanisms triggered by the intervention and (2) to develop the flow and content of the intervention. During this phase, a decision was made on the target behaviors of the intervention. The behavior change wheel (BCW) method was used to analyze the target behaviors and to design intervention components [41]. First, based on the literature, behavioral analysis was performed by two researchers (YK and SH) to identify what needs to change in patients’ capability, opportunity, and motivation to improve each target behavior (capability, opportunity, and motivation model of behavior [COM-B] analysis) [41]. Second, the theoretical domains framework (TDF) was used to elaborate on the behavioral analysis by mapping the 14 domains of the framework onto the capability, opportunity, and motivation components of COM-B [41]. Third, potentially relevant intervention functions and behavior change techniques (BCTs), matching users and context, were selected using criteria provided by the BCW [41] (see Multimedia Appendix 1). Logic modeling was used to map out the potential working mechanism of the intervention by detailing all evidence and assumptions underpinning the pathway from the intervention to the long-term impact on outcomes [42,43]. The logic model starts with the target behaviors and details what needs to change in behavior (TDF), by which intervention functions and BCTs, and through which specific intervention components, including factors that could influence the working mechanism, and results in short- and long-term outcomes. The logic model components were based on the evidence gained from all previous phases, and consensus on the components was reached during research group meetings. On the basis of this model, design requirements were formulated.

Furthermore, creative ideas with regard to the intervention design were explored using methods derived from design thinking [44]. In a *pressure cooker session* with three independent creative designers, initial ideas on the design were presented by focusing on potential techniques to change health behaviors and to enhance engagement with mHealth. After this session, collaboration with a creative design agency (Panton BV, Deventer, the Netherlands) specifically focusing on health care solutions was initiated. By following an iterative design process, the flow and content of the intervention were designed, and various design styles were developed using low-fidelity prototypes—paper prototypes that visualize design solutions. In the early stages of digital user interface design, such low-fidelity paper prototypes are often used to determine requirements for the architecture and functionalities of the specific intervention to be designed [45]. The paper prototypes were tested in phase 2 of the UCD.

Moreover, the content of a symptom-monitoring module was developed during this stage. The module aimed to determine the individual COPD patient’s normal day-to-day variability in symptoms to be able to set the patient’s normal symptom pattern. The content validity of the module was evaluated by experts in the field of COPD (n=8) according to the Lynn method [46]. Each symptom was rated on *relevance* and *linguistics* by answering four questions*.* All questions about *relevance* were rated on a 4-point Likert scale (1=not relevant, 4=relevant). *Linguistics* was determined by if the interpretation was clear (*yes* or *no*). The item-content validity index (I-CVI) was calculated for each *relevance* question to determine the number of experts judging the content as valid (I-CVI>0.78=relevant). Subsequently, the scale-content validity index (S-CVI) was calculated to determine the relevance of the whole symptom-monitoring module (S-CVI>0.90=excellent) [46]. *Linguistics* was considered to be clear when at least 75% of the expert panel rated clearness of interpretation as a *yes*. A more in-depth description of the development and content validity assessment of the symptom-monitoring module is given in Multimedia Appendix 2.

#### Phase 2: Alpha Usability Testing

In the second phase, alpha usability tests were performed by investigating patient and HCP responses to low-fidelity paper prototypes of the intervention in two steps: (1) evaluating the intervention flow and content and (2) evaluating intervention design styles [33]. At each phase of usability testing, we only included patients who had not evaluated an earlier prototype.

##### 2A: Evaluate Intervention Flow and Content

Perceptions, needs, and preferences regarding the intervention flow and structure were evaluated with both patients with COPD (n=6) and HCPs (n=6) to identify usability requirements (Figure 1; phase 2A). Individual semistructured interviews were held using low-fidelity paper prototypes. The following topics were discussed: experience with mHealth and written action plans, the overall flow of the intervention, symptom-monitoring/action plan scenarios, and the added value of the intervention. Purposive sampling of participants was performed in primary, secondary, and tertiary care settings. In total, 6 patients and 6 HCPs were included based on the general rule of thumb that approximately 80% of all potential usability problems could be identified by including 5 to 10 end users [47]. The inclusion and exclusion criteria of the participants during usability testing are detailed in Textboxes 2 and 3, respectively. Data were thematically analyzed by two researchers independently [48]. Data analysis was supported by NVivo 10.0 software (2012; QSR International Pty Ltd.).

**Inclusion criteria of participants during usability testing.**

**Inclusion criteria for patients with a clinical diagnosis [2] of chronic obstructive pulmonary disease (COPD)**

- Age >40 years
- Spirometry forced expiratory volume in 1 second/forced vital capacity ratio <70%
- ≥1 exacerbation in the last 12 months before entering the study (defined as a period of symptom deterioration in which the use of a course of corticosteroids and/or antibiotics was required, or hospitalization was necessary)
- Adequate communication skills
- Willing and able to comply with study procedures and give written informed consent
- Patients who are judged by their health care provider to have suitable hearing and vision

**Inclusion criteria for health care providers**

- Having a patient–health care provider relationship with patients with COPD
- Supporting patients with COPD in self-management
- At least one year of experience with COPD care

**Exclusion criteria of participants during usability testing.**

**Exclusion criteria for patients with a clinical diagnosis of chronic obstructive pulmonary disease**

- Diagnosed with cognitive impairments
- Life expectancy ≤3 months
- Primary diagnosis of asthma, cardiac disease, or other major functionally limiting diseases

**Exclusion criteria for health care providers**

- Not applicable

##### 2B: Evaluate Intervention Design Style

Next, the preferences of patients with COPD regarding intervention design style were explored by individual semistructured interviews (n=11; Figure 1; phase 2B) [33]. Low-fidelity paper prototypes were used to present variations in design style and tone of voice. Purposive sampling of participants was performed in a physiotherapy practice and a rehabilitation center according to the inclusion and exclusion criteria for patients in Textboxes 2 and 3. Data were analyzed by 2 researchers independently through summarizing the advantages and disadvantages of each design style and the overall preferences regarding design style. On the basis of the results of both alpha usability steps, the intervention design was finalized for further software development.

#### Phase 3: Iterative Software Development

The software of the mHealth intervention was developed during a 12-week period according to a scrum-based design method consisting of five development sprints (Figure 1; phase 3) [49]. During biweekly stakeholder meetings, the research team, designers, and software developers met in person to evaluate the current stage of development and to make decisions on the further development of the first version of the mHealth intervention (minimum viable product; MVP). The mHealth intervention was built in React Native (Massachusetts Institute of Technology licenses), a software structure that is easy to adapt and suitable for both iOS and Android. This saves time and money during the initial and future development of the intervention and fits within the agile development process of the intervention.

#### Phase 4: Field Usability Testing

In the fourth phase, field usability tests of the MVP (ie, tests with a high-fidelity prototype within the context in which the intervention will actually be used) were performed with patients with COPD (n=7) and HCPs (n=3) using cognitive task analysis [47,50]. This mixed methods study focused on three quality components: task success, user errors/problems, and satisfaction, based on Nielsen’s heuristics and the International Organization for Standardization’s usability standard 9241-11 [51]. Purposive sampling of participants was performed in primary, secondary, and tertiary care settings until data saturation was reached. In line with the procedure of phase 2, a minimum of 5 patients were included according to the inclusion and exclusion criteria of Textboxes 2 and 3. Participants were observed while performing tasks with the MVP and asked to *think aloud* to clarify their decision-making process and express experienced user problems and errors [51]. After the task analysis, the validated 10-item system usability scale (SUS) was filled out by patients to get a global view of usability [52]. Each item was scored on a 5-point Likert scale, and all items were converted to a total score (range 0-100, a score>70 is considered to be acceptable) [52,53]. Furthermore, semistructured interviews were conducted. On the basis of previous research and the technology acceptance model [54,55], the following topics were formulated: the first impression of the app, ease of use, satisfaction, perceived usefulness, applicability, attitude toward using the app, and the content of the app. The whole procedure with patients was video recorded without the faces of participants being visible. The procedure with HCPs was more pragmatic in nature because the MVP did not include a specific HCP interface. However, the relevant functions for HCPs could be tested within the MVP. Therefore, only 3 HCPs were included, and the procedure was only observed by 1 researcher who simultaneously made notes.

The performance of tasks by patients was observed by two researchers independently. An observation list was used to note task success, users’ errors/problems, and participants’ expressions for each task. The performance of tasks was scored as successful (1 point), partially successful (0.5 points), or unsuccessful (0 points) [56]. The observation lists were discussed by the researchers to reach a consensus on the performance of tasks and the identified problems and errors. The data from the think-aloud method were used to derive a better understanding of task performance. A severity score ranging from 0 (no usability problem) to 4 (usability catastrophe) was given to each problem based on the impact and frequency of the problem [57]. Data from the semistructured interviews were analyzed by 2 researchers independently using thematic analysis [48]. The data analysis of HCPs observations was performed by only 1 researcher, and the semistructured interviews were only summarized.

The usability studies were approved by the Medical Ethics Research Committee of the University Medical Center Utrecht (17–887), and all participants gave written informed consent.

### Business Modeling

Business modeling, based on the principles of *the lean startup* methodology [58], was performed parallel to phase 1F until phase 4 to ensure valorization and sustainable implementation of the mHealth intervention in its intended care practice (Figure 1) [59]. Business modeling included contextual inquiry and continuous investigation of relevant stakeholder needs (patients with COPD, HCPs, policy makers, and health care insurers) to better understand what should be accomplished with our mHealth intervention and to obtain value drivers to underpin choices in what to design [59]. The needs of patients with COPD and HCPs were investigated in phases 1C, 1D, and 1E, and individual conversations with policy makers and health care insurers were held to identify their perspectives toward the mHealth intervention. Furthermore, the best innovation and distribution routes and market opportunities were explored in conversations with stakeholders to investigate their interests and financial incentives to support self-management with mHealth. Competition analysis was performed to explore the value of our intervention with respect to existing mHealth technologies. Finally, conversations with vendors in the field were held to explore business opportunities.

## Results

### Phase 1: Background Analysis and Design Conceptualization

#### 1A: Identify the Evidence Base

A total of four relevant systematic reviews on exacerbation-related self-management interventions and two systematic reviews specifically focusing on mHealth interventions to improve exacerbation-related outcomes were identified. Self-management interventions, including exacerbation action plans along with ongoing self-management support, were associated with positive outcomes on quality of life, hospital admissions, and health care use [11,12,60]. A review of self-management interventions delivered immediately following an acute exacerbation showed no significant effect on quality of life nor hospital admissions [61]. All reviews showed large heterogeneity in interventions making it hard to draw conclusions on effective components of these interventions. Furthermore, mHealth interventions facilitating, supporting, and sustaining self-management among people with COPD significantly improved quality of life, and levels of activity [19]. Smartphone interventions in patients with COPD with exacerbations, without a specific focus on self-management, were found to be useful in reducing the number of patients having a COPD exacerbation [20]. These results should also be interpreted with caution because of the heterogeneity among studies.

On the basis of these findings, it seemed promising to use mHealth strategies that specifically aim at enhancing self-management behavior. It was considered important that the mHealth intervention includes a COPD action plan along with ongoing self-management support, which confirmed our guiding principle to include an action plan. Furthermore, a conceptual definition of a COPD self-management intervention was published in 2016 [16]. Given the need for consensus on what defines a COPD self-management intervention, this definition was added to the guiding principles (Textbox 1).

#### 1B: Identify Key Behaviors and Concept Mapping

A conceptual model picturing the event of an exacerbation was developed by distinguishing five phases before, during, and after an index event. Specific aims regarding the reduction of exacerbation impact were formulated for each phase of the conceptual model. The conceptual model is published elsewhere [15]. On the basis of the knowledge generated from the literature, an initial set of 27 relevant self-management behaviors aiming to reduce exacerbation impact was identified and assigned to the relevant phases of the conceptual model. This initial set of self-management behaviors was introduced to experts in the first round of the Delphi study (Figure 1; phase 1C) to reach a consensus on the most relevant behaviors [15].

#### 1C: Problem Analysis

A Delphi panel of 19 international experts reached a consensus on 17 self-management behaviors that can be targeted and influenced before, during, and after an exacerbation (Figure 1; phase 1C). This set of behaviors has the potential to maximally reduce the impact of exacerbations. The self-management behaviors were related to the following broader categories: adherence to pharmacotherapy, influenza vaccination, physical activity/exercise, avoiding stimuli, smoking cessation, early detection of symptom deterioration, medical treatment of exacerbations, managing stress and anxiety, and awareness of recurrent exacerbations [15]. The 17 self-management behaviors were considered as potential target behaviors for the mHealth intervention. Our grounded theory study (Figure 1; phase 1C) has resulted in a conceptual model explaining factors that influence exacerbation-related self-management from the patients’ perspective. The conceptual model is published elsewhere [30]. The conceptual model shows that exacerbation-related self-management is influenced by five generic factors: *acceptance of COPD, perceived severity of symptoms, knowledge of exacerbations, former experiences with exacerbations*, and *social support*. Furthermore, *heterogeneity of exacerbations* and *habituation to symptoms* were identified as specific factors influencing the capability to recognize an exacerbation. Performance of self-management actions was specifically influenced by *perceived influence on exacerbation course, feelings of fear, self-empowerment, trust in health care provider, patient beliefs*, and *ambivalence toward treatment* [30]. These factors were included as moderating and mediating factors in the working mechanism of the intervention (see also 1F: Intervention Design section).

#### 1D: Needs Analysis

Our needs analysis (Figure 1; phase 1D) resulted in an overview of potential benefits and barriers regarding the use of mHealth to support self-management and early ideas on the content of the intervention [31]. Both patients and HCPs emphasized the need for a multicomponent and tailored mHealth intervention that focuses on improving patient self-management skills by determining health status and providing adequate information, decision support, and feedback on self-management behavior in an advisory manner. Important findings were that patients and HCPs emphasized that an mHealth intervention should never replace patients’ own feelings nor undermine their own decisions. The intervention should be complementary to regular (personal) contact with HCPs and should facilitate adequate self-management support by HCPs. Discussing self-management skills with HCPs in personal consultations was believed to be essential to improve these skills. Both patients and HCPs expressed doubts regarding (real-time) the monitoring of symptoms by HCPs because of safety reasons and time constraints, although early detection of exacerbations was considered to be an important benefit. Moreover, the intervention should be attractive, straightforward, rewarding, and safe. Finally, patients emphasized that using mHealth should be their own choice and should never be enforced. On the basis of these findings, the design requirements for the intervention were formulated. Further results of the focus group interviews are published elsewhere [31].

#### 1E: Current Practice Analysis

On the basis of three Dutch health care standards focusing on COPD [62-64] and 10 interviews with HCPs, insight into current exacerbation-related self-management support was provided. Two pulmonologists, 2 nurse specialists, 2 pulmonary nurses, 2 general practitioners, and 1 primary care nurse (4 males/6 females, work experience range 3-20 years) were interviewed. An important finding was the lack of standardized self-management support and limited use of evidence-based interventions by HCPs. There was a large variation in providing information about exacerbation-related self-management with regard to timing, topics discussed, and mode of delivery. Only a few HCPs used a COPD action plan and prescribed self-treatment with prednisolone and/or antibiotics to stimulate self-management. More than half of the HCPs (n=6) expressed that patients had no specific case manager. Providing self-management support was mostly perceived as a shared responsibility between HCPs, although individual responsibilities of the HCPs involved were unclear. Most HCPs felt that there is large room for improvement in self-management support by HCPs. Barriers in providing self-management support were the lack of standardized self-management support and clarity in responsibilities between HCPs, limited availability of HCPs, limited case management, limited time during consultations, limited financial resources, and suboptimal interdisciplinary communication in COPD care. The findings from the current practice analysis were included as moderating and mediating factors in the working mechanism of the intervention (see also 1F: Intervention Design section).

#### 1F: Intervention Design

**Figure 2:**
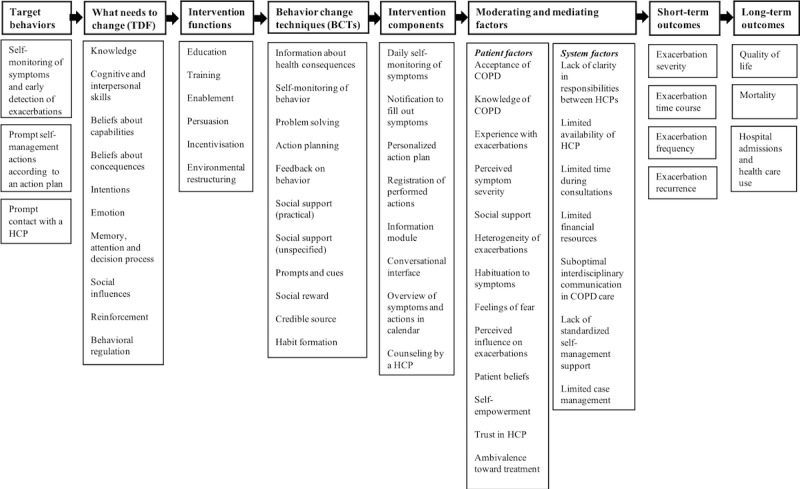
Logic model of a mobile health intervention to enhance exacerbation-related self-management in patients with chronic obstructive pulmonary disease. BCTs: behavior change techniques; COPD: chronic obstructive pulmonary disease; HCP: health care provider; TDF: theoretical domains framework.

During the intervention design phase, the research team decided to initially focus on the three target behaviors: (1) self-monitoring of symptoms and early detection of an exacerbation, (2) taking prompt individualized self-management actions, and (3) prompt contact with an HCP. On the basis of insights from phase 1C, these behaviors were expected to contribute most to the reduction of exacerbation impact, had the potential for large improvement, and were considered most feasible to influence. The choice for these three behaviors was made, given the importance of aggregating the target behaviors that fit together and are considered to have the largest impact on exacerbations [65]. On the basis of behavioral analysis of these behaviors, potential intervention functions and BCTs were selected for the intervention [41]. The behavioral analysis of the target behaviors, including the final intervention functions and BCTs is described in detail in Multimedia Appendix 1. Figure 2 shows the logic model of the intervention that synthesizes all the evidence gained in the previous phases (phase 1A until 1E), including the selection of final intervention functions and BCTs.

On the basis of the results of all previous phases, design requirements for the mHealth intervention were formulated (see Textbox 4). At this stage, the research team and design agency decided to develop a mobile app to enhance exacerbation-related self-management in patients with COPD. On the basis of design requirements, a concept of the flow and content of the app and various design styles were developed using low-fidelity paper prototypes.

**Design requirements for the mobile health intervention.**

The mobile health (mHealth) intervention should:

- at least focus on self-monitoring of symptoms and early detection of exacerbations and taking prompt self-management actions including prompt contact with a health care provider (HCP);
- support patients in developing self-management skills over time (*learning by doing*) and changing behaviors;
- focus on (aggregated) self-management behaviors before, during, and after an exacerbation;
- include a chronic obstructive pulmonary disease (COPD) action plan with an educational component along with ongoing support;
- be comprehensive/multicomponent and tailored to individual patients;
- provide adequate information, decision support, and feedback on self-management behavior (in an advisory manner);
- take into account the factors influencing exacerbation-related self-management;
- fit with current COPD care and be accessible for HCPs;
- facilitate adequate self-management support by HCPs;
- be complementary to regular (personal) contact with HCPs;
- be attractive, straightforward, rewarding, and safe;
- never replace patients’ own feelings nor undermine their own decisions; and
- not be enforced to patients. Using mHealth should be the patient’s own choice.

Furthermore, the symptom-monitoring module was developed during this stage. Content validity of the symptom-monitoring module was determined after two expert rounds. In total, these eight symptoms were rated as relevant (I-CVI>0.78) and clear (≥75% of the expert panel): Dyspnea, wheezing, nighttime symptoms, coughing, sputum volume, sputum purulence, sputum color, and fatigue. The relevance of the final symptom-monitoring module, determined by three questions, was considered to be high with S-CVIs of 0.93 or greater. More detailed results and the final symptom-monitoring module are shown in Multimedia Appendix 2.

### Phase 2: Alpha Usability Testing

#### 2A: Evaluate Intervention Flow and Content

Evaluation of the flow and content of the intervention with both patients with COPD and HCPs resulted in overarching themes related to the intervention flow and an overview of usability requirements. The baseline characteristics of the participants are shown in Multimedia Appendix 3. The patients recruited from tertiary care all had a written action plan, whereas the other patients only had verbal agreements with their HCPs.

##### The Intervention Flow and Content

**Figure 3:**
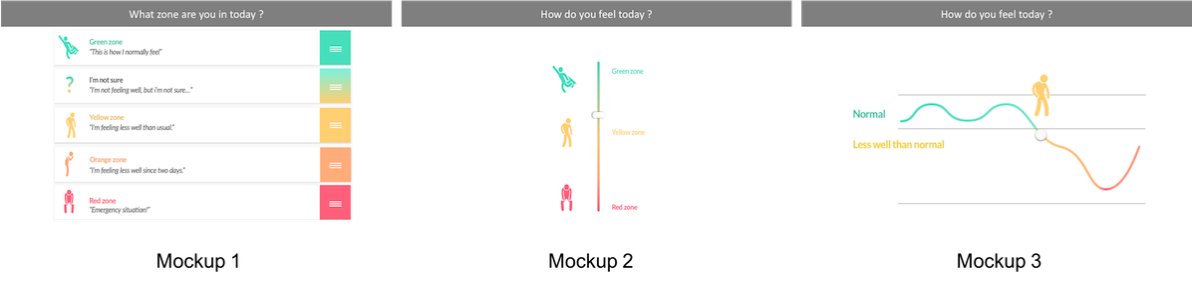
Low-fidelity paper prototypes of 3 scenarios to determine symptoms status.

Overall, all patients and HCPs were positive about the intervention flow that consisted of four steps: (1) personalization of an action plan, (2) intensive monitoring of symptoms, (3) adjusting initial action plan based on monitoring period, and (4) regular use (filling out symptoms on a regular basis and receiving support on individualized actions). However, the patients who believed that they were well aware of their symptoms did not directly perceive that they could benefit from the intensive monitoring period. Overall, patients preferred personalization of the duration of intensive monitoring and the timing of notifications. HCPs felt that they should have autonomy in determining how, and at which moment, the action plan should be reviewed and adjusted. On the basis of specific mockups used to explore preferences regarding symptom registration and determining symptoms status, the most intuitive and straightforward scenarios were identified. For example, mockup 1 was considered to be the best solution to determine symptom status by all patients (Figure 3).

##### The Added Value of the Intervention

Patients expressed that the app could create awareness into their own situation and could support early detection of symptom deterioration and taking prompt actions. The adjustability and accessibility of the app were perceived as benefits compared with using a written action plan. Furthermore, both patients and HCPs were positive about the overview of registered symptoms and undertaken actions as a tool to start the dialog about patients’ self-management behavior. Moreover, personalization and tailoring of the app were considered to be an important benefit:

Finally an app that is not for COPD but for me personally.

Patient 2

##### Needs With Regard to the Intervention

Patients expressed a clear need for an accessible and reliable app that provides insight into their own situation and eliminates their doubts by including reflective questions. Patients stressed the importance of an app that is straightforward, for example, by providing simple and effortless instructions in case of serious dyspnea that causes panic, such as:

breathe slowly or call the doctor: It has to be simple, because energy is air.

Patient 3

They would like to use the app to inform relatives about their situation. Both patients and HCPs emphasized that the app should stimulate prompt contact with an HCP:

Patients experience feelings of fear you know, like: when I am raising an alarm, I might have to take prednisolone or I might be admitted to the hospital, so therefore I won’t make the call...Will that be included in the app as well?

HCP 1

Furthermore, HCPs explained that the app should provide insight into patient symptoms over time and realize more proactive care instead of reactive care. Most HCPs felt that a separate HCP interface to personalize the app would increase the usability of the app in daily practice.

##### Usability Requirements

On the basis of the results of phase 2A, usability requirements were formulated for the software development phase (see Table 1).

**Table 1:** Usability requirements for the mobile health intervention.

Topics of importance to users	Patients with chronic obstructive pulmonary disease (n=6) and HCPs^a^ (n=6)
Content	App should be reliable and accessible Information should be straightforward and individualized App should provide insight into symptoms over time App should support prompt contact with an HCP App should be a tool that can be used to get into dialog with HCPs about self-management behavior
Tailoring of the app	Intensive monitoring period and timing of notifications should be personalized HCPs should have autonomy in determining how, and at which moment in the care process, the action plan should be reviewed and adjusted
Interface	The action plan should preferably be personalized in a separate HCP interface
Design style	The design style should be restrained and clear without too much text.

^a^HCP: health care provider.

#### 2B: Evaluate Intervention Design Style

**Figure 4:**
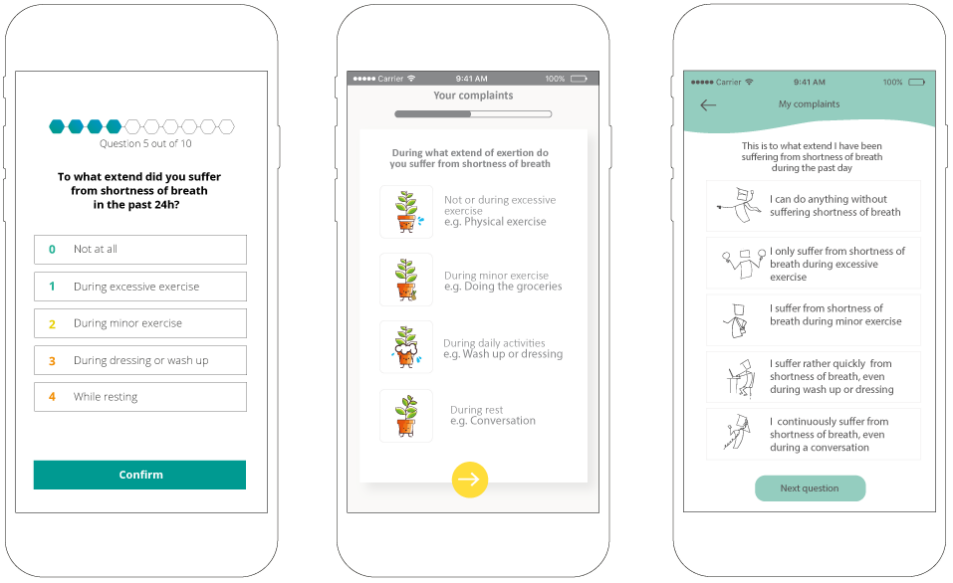
Low-fidelity paper prototypes of three symptom monitoring design styles.

In total, three potential design styles of the intervention were explored with patients with COPD (see Baseline characteristics in Multimedia Appendix 3. Mockups of the action plan, symptom registration, action registration, and the overview of symptoms and actions over time were used (see example in Figure 4). There was no consensus on a preferred design style. In general, patients preferred a restrained and clear design style without too much text. A few patients were positive about a more numerical design, whereas other patients found it hard to express their symptoms in numbers. There was a wide variety in preference regarding the tone of voice (distant vs personal tone of voice). Overall, patients were positive about using symbols. Most patients were negative about using an avatar in the app as it has no added value, and some patients considered an avatar to be childish. On the basis of these results, a restrained and clear design style without too much text was included as a usability requirement (see Table 1).

### Phase 3: Iterative Software Development

Iterative software development resulted in a functional mobile app for patients (Copilot app) that can be used to (1) compose an action plan together with an HCP (based on a COPD action plan using color zones that is included in Dutch care standards)[66], (2) monitor symptoms and undertaken actions, (3) review symptoms and undertaken actions, and (4) read information about COPD and exacerbations. At this stage, the final integrated list of BCTs needed to ensure this MVP adhered to both the guiding principles (Textbox 1) and the design requirements (Textbox 4) was constructed by two researchers (YK and SH) using the BCW framework. In total, 6 intervention functions and 11 BCTs were selected for the MVP (see Figure 2 and Multimedia Appendix 1). During the iterative software development process, the research team made decisions to add steps to the flow of the app that were not thought of beforehand, such as including an onboarding program to register and personalize the patient’s action plan. At the same time, owing to time and financial constraints, some steps were disregarded and moved to later versions of the intervention, such as including assistance in cases where patients are in doubt about contacting their HCP.

### Phase 4: Field Usability Testing

**Figure 5:**
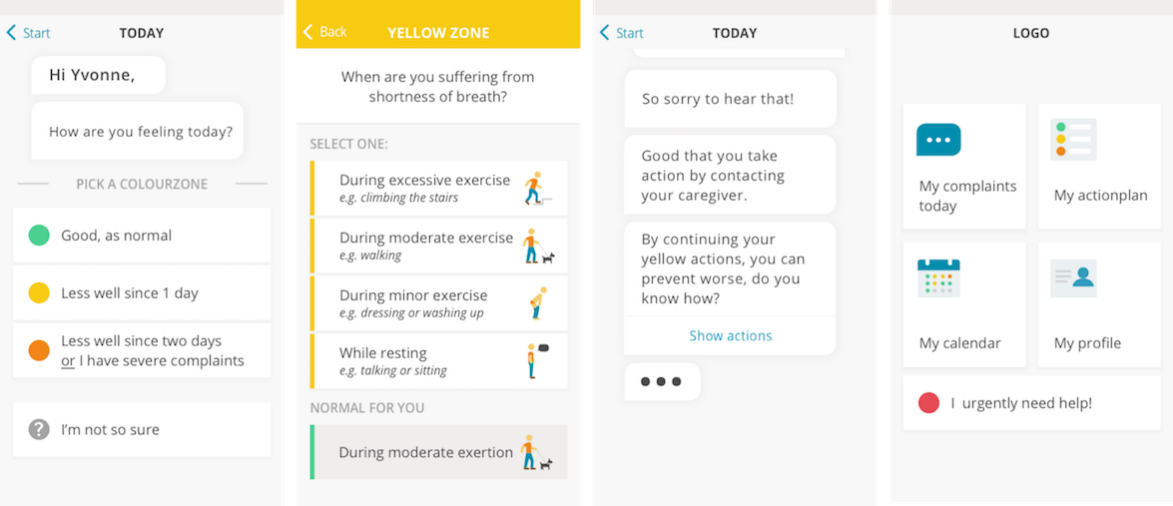
High-fidelity prototype of the Copilot app (minimum viable product).

All functionalities of the MVP were tested by patients with COPD, and all functionalities that belong to the HCP role were evaluated with HCPs (see baseline characteristics in Multimedia Appendix 3). Examples of the MVP are shown in Figure 5.

#### Usability Assessment

According to patients with COPD, the general usability of the MVP was considered to be good based on the average rating of 90.7 (SD 6.7) on the SUS.

#### Task Success

Key tasks within the app were performed by all patients, and some tasks were performed only by a few patients because of time constraints (see Multimedia Appendix 4). Almost all patients were successful in consulting the app for HCP contact details and immediate help as well as to check self-management actions in the specific color zones. Furthermore, reviewing symptoms and undertaken actions was performed successfully overall, except for 2 participants who did not know where to find this overview. The information module was easily found by most of the patients. Most difficulties in the performance of tasks were observed during the symptom and action registration. Most patients were able to fill out their symptoms, but some patients overlooked the *save* button. One patient experienced difficulty with selecting the right color zone because of difficulty with scenario thinking. The support option for selecting a color zone (gray zone) appeared not to be intuitive, and navigation problems in the gray zone were observed. Overall, the HCPs were able to personalize the action plan and to evaluate the symptom-monitoring period. The performance of tasks by both patients and HCPs is further specified in Multimedia Appendix 4.

#### User Errors and Problems

On the basis of task analysis and observations during random navigation in the app, 23 user errors and problems were identified by patients with COPD. In total, seven problems were rated with the highest severity score of 4. These problems were related to saving registered symptoms, accidentally deleting symptoms and navigation in the gray zone. Moreover, five problems had a severity score of 3 and were related to the action plan overview, understanding of buttons on the home screen, and changing HCP contact details. The lowest severity scores (1 and 2) were assigned to 11 problems. Furthermore, 11 user errors and problems were observed during the use of the app by HCPs. The HCPs experienced problems with saving registered symptoms as well. Another severe problem was related to changing contact details (severity score 4). Two less severe problems were related to personalizing and changing the yellow zone of the action plan (severity score 3). The lowest severity scores (0-2) were assigned to seven problems. A more detailed overview of all user problems and errors is provided in Multimedia Appendix 4.

#### Patients’ and Health Care Providers’ Perceptions Toward Using the App

Overall, patients were positive about the app, as they found the app supportive with regard to monitoring and evaluating symptoms and taking prompt actions. On a scale from 1 to 10, patients’ satisfaction rates ranged from 8 to 10 because of the ease of use and the interface being intuitive. The patients who frequently experienced exacerbations expressed an important need for the app. Two patients who rarely experienced exacerbations explained that the app will only have an added value if additional support would be provided during the stable phase (green zone). The interviews resulted in 13 themes that were categorized into feelings about the app, the added value of the app, the content of the app, and facilitators and barriers to use the app. A description of these themes and illustrative quotes is provided in Multimedia Appendix 4.

Field usability testing showed that a usable mHealth intervention has been developed. On the basis of these results, improvements for future versions of the app were revealed. The improvements were determined in collaboration with the designers and focus on the user errors and problems that were rated as most severe (severity ratings 3 and 4), problems that were observed in both patients and HCPs, or additional problems that were mentioned in the interviews. An overview of these improvements is shown in Multimedia Appendix 4.

### Business Modeling

Business modeling resulted in a preliminary business plan that provided important design input during the development steps and will direct future development and implementation steps. The business plan guided the direction to actively involve HCPs in providing the intervention to patients and the specific focus on developing self-management skills over time (learning by doing) to be distinctive from other Dutch mHealth solutions. These outcomes were added to the design requirements (Textbox 4). During business modeling, market volume, and segmentation, different innovation and distribution routes and revenue models were systematically evaluated. Given current positive developments with regard to funding of apps in the Netherlands, especially those that are evidence-driven, a distribution strategy will be chosen that includes health care insurers to ensure implementation and continued use in Dutch COPD care.

## Discussion

### Principal Findings

This paper provides insight into a systematic and thorough way of developing an evidence-driven and usable mHealth intervention (Copilot) for patients with COPD to enhance exacerbation-related self-management. Following an iterative UCD process, a mobile app consisting of a personalized action plan and symptom-monitoring module has been developed. The intervention was developed by following a thorough and well-underpinned process consisting of a background analysis and design conceptualization phase leading to final guiding principles, a logic model and design requirements, usability testing phases leading to usability requirements, and iterative software development of an MVP that adheres to these guiding principles and design and usability requirements. This unique approach of scientific engineering has resulted in an mHealth intervention that meets the needs and preferences of patients with COPD, is likely to be used by patients with COPD, and has a high potential to be effective in reducing exacerbation impact. Involving patients with COPD, HCPs, COPD experts, and experts from design and behavioral science throughout the development process increased the likelihood that the mHealth intervention can be successfully implemented into Dutch COPD care.

Copilot requires an active case manager role, as previous studies have shown the need for ongoing case manager support alongside the use of an action plan to achieve effective and safe self-management [11,67]. The mHealth intervention was developed as a tool to enhance patient self-management skills that is complementary to personal interaction with an HCP. This is in line with recent research underlining that a good patient-HCP relationship is important for patients to engage and take responsibility for their own health care [68,69]. The specific focus on developing self-management skills over time is distinctive from other mHealth initiatives, as research in the past decade has focused increasingly on telemonitoring strategies to decrease the impact of exacerbations [21,70,71]. The impact of telemonitoring in the COPD population is, however, still equivocal because of trial designs, unstandardized interventions, and limited follow-up [21,70]. With telemonitoring, the decision-making process is profession based. The working mechanism of Copilot focuses on enhancing patients’ self-management skills over time. Therefore, no telemonitoring strategies were included in our mHealth intervention.

Although our Delphi study has shown the need for a comprehensive strategy to improve the full spectrum of exacerbation-related self-management behavior [15], the first version of our mHealth intervention focuses specifically on self-monitoring of symptoms and taking prompt individualized self-management actions. A *less is more* approach consisting of only a few strong target behaviors that fit together was considered to be imperative in creating impact [41,65]. When changing these target behaviors is proven effective, we could build upon these behaviors incrementally [41]. It is important to note that not all relevant self-management behaviors have to be addressed through mHealth, as HCPs should continue to have an essential role in providing self-management support as well [31].

### Strengths and Limitations

A major strength was the systematic and thorough way of developing Copilot according to a UCD that was based on existing development models and diminished the chance of missing important steps [33,34]. We have systematically investigated and incorporated the views of end users, continuously evaluated prototypes, and involved persuasive design techniques to match user profiles and motivate patients to engage in self-management, which is in line with the *person-based approach* and the holistic framework for the development of electronic health (eHealth) technologies [32,72]. Furthermore, we used guiding principles to easily recall the principal and distinctive features of the intervention during the extensive, iterative intervention development process [32]. Another important strength was the detailed analysis of behaviors of patients with COPD using the BCW method and selection of BCTs to underpin the pathway toward behavior change [41]. Using the BCW method along with a UCD is comparable with the methodology used by Curtis et al [22] to develop a theory-driven and user-centered healthy eating app. Their work also focused on a thorough analysis of target behaviors, selection of BCTs, and exploration of user preferences to underpin the design of the app with relevant theory and evidence and ensure engagement among the target population. However, Curtis et al [22] performed no specific activities with regard to valorization and implementation of their app during their development process [22]. To make both the design and the implementation of our app value driven, we performed valorization activities throughout the development process of our app [59,72]. Business modeling helped us to identify critical success factors that will influence the sustainability and effectiveness of the app, which is often overlooked during the development process of eHealth and mHealth technologies [59].

From a health care and behavior change perspective, we chose to use the MRC framework for the development of complex interventions as a basis for our UCD, instead of a more general software development approach. The four iterative phases were inspired by the user-centered methodology used by Johnston et al [33] for the development of a Web-based interface for patients with COPD. The use of a more general software development approach as a basis for the development process might have provided more specific guidance to the software development and usability phases beforehand. However, such approaches pay less attention to the activities needed to design a theory- and evidence-driven intervention, which was an important focus in our design process.

Furthermore, it should be noted that the extensive and thorough development process increased the likelihood of developing an effective intervention, although it is questionable if this process is feasible in daily practice. The whole intervention development process took place over a 4-year period, which is quite time consuming and could increase the risk of a misfit with current market developments or that technology has moved on by the time of implementation [22,73,74]. The time-consuming development was partly because of the inclusion of all the development phases but was also related to developing an mHealth intervention from a scientific environment. Developing an intervention from science involves completing an empirical cycle at each development phase and often includes an extensive review of a study protocol by a medical ethics research committee. Pursuing the rules of science during the development process of an mHealth intervention has slowed down the process at certain points in time. Furthermore, development from a scientific environment generally means less focus on business modeling and entrepreneurship, which could delay the process of bringing the app to the market. Finally, a limitation in this study was the restricted budget available for the creative design and development of the mHealth intervention, which required us to make a selection in the development of intervention components.

### Implications for Practice and Future Research

The findings of this study are important for both patients with COPD and HCPs supporting patients with COPD in self-management as well as for researchers and designers focusing on the development of mHealth interventions. For patients with COPD, an evidence-driven and usable mobile app has been developed to assist them in developing exacerbation-related self-management skills. It needs to be emphasized that, at least for the coming years, not all patients with COPD will be eligible for the mHealth intervention, especially for those with a more negative attitude toward mHealth and low digital literacy [19,31]. However, a positive change in attitudes toward mHealth and digital skills can be expected in the future, given the current trends in internet access and smartphone use [18]. For HCPs, the mHealth intervention can be used to provide more evidence-based, structured, and tailored self-management support. The mHealth intervention can be embedded in primary, secondary, and tertiary care settings, which could contribute to improving integrated care. To increase the likelihood of successful implementation in Dutch COPD care, the intervention can easily be adapted to a specific setting and context. Hereby, the intervention would be available for a wide range of health care settings in which patients with COPD are currently treated. For future practice, it is important that more intervention components will be added to the mHealth intervention to optimally address the selected target behaviors, such as adding self-treatment with prednisolone and/or antibiotics and providing assistance in case patients are in doubt about if they should contact their HCP. Furthermore, a separate dashboard for HCPs should be developed to be able to individualize the mHealth intervention and to review registered symptoms and actions during consultations without having to use the patient’s own device. An essential step would then be to establish cooperation with external vendors in the field and health care insurers to ensure implementation in COPD care. In the next phase, it is important that the mHealth intervention takes into account patient comorbidities to make the intervention available for a wider population and to ensure patient safety [12]. Future steps should focus on adding target behaviors that are relevant before, during, and after an exacerbation to maximize the reduction of exacerbation impact.

For researchers and designers, the UCD in this study can be used as guidance for the development of mHealth interventions that meet end user needs and preferences, have high potential to be effective, and are likely to be used by the target population. Essential in the development is that interventions are grounded in theory and evidence and that user needs and preferences are thoroughly investigated. Moreover, valorization and implementation activities should be regarded as continuous activities throughout the development process to ensure sustainable use in its intended practice. This extensive reporting of the intervention development process enhances the reproducibility of the intervention and contributes to more transparency in the development of complex interventions in health care, which is needed to strengthen the internal and external validity of interventions and to add value to health care research [34]. All in all, it is helpful to have multiple examples and variants on how to develop evidence- and theory-driven mHealth interventions. It should be considered if the thoroughness of this UCD is needed for all mHealth interventions that will be developed in the future. Depending on the topic, decisions should be made about which phases and steps are relevant to the topic and should be included in the development process. In addition, taking time aspects into consideration, it should be questioned how thoroughly an individual step should be executed. The need for efficiency in the development of mHealth interventions is currently a highly discussed topic [75]. Our work contributes to this discussion by mapping out a state-of-the-art design and development process and showing how time consuming this is.

Future research should focus on evaluating the feasibility of the mHealth intervention in the daily practice of HCPs, as they have a key role in personalizing the mHealth intervention before patient use. In a second phase, the feasibility of the mHealth intervention should be evaluated with patients with COPD to investigate the delivery and acceptability of the intervention, compliance with the intervention, and recruitment and retention of patients. In the next phase, the effect of the mHealth intervention on the relevant patient outcomes and health care use should be evaluated. Recent studies on mHealth interventions in patients with COPD suggest the use of randomized controlled trials (RCTs) with adequately powered sample sizes and a 1-year follow-up period to be sufficient to comment on behavioral change and impact of treatment [19,20]. However, this time-consuming design may not be ideal for rapidly evolving mHealth technologies [73,76]. Using an RCT implies two or more years of research in which this mHealth intervention with high potential for effectiveness, and no expected harm will not be available for patients with COPD. Furthermore, an RCT only enables identifying if this complex mHealth intervention as a whole work and the cost-effectiveness of it, without identifying which intervention components work in whom. Alternatively, more rapid study designs such as n-of-1 trials or observational designs could be used to understand the working mechanism of the intervention and simultaneously focus on bringing the mHealth intervention to the market as soon as possible [73,75-77]. Within these designs, it is important to evaluate self-management skills and behavior change as outcomes, and the way this is assessed should be clearly reported [19,77,78].

### Conclusions

This paper described in detail the full UCD and development process of an evidence-driven and usable mHealth intervention to enhance exacerbation-related self-management in patients with COPD. By following a UCD process, an mHealth intervention was developed that meets the needs and preferences of patients with COPD, is likely to be used by patients with COPD, and has a high potential to be effective in reducing exacerbation impact. This extensive reporting of the intervention development process contributes to more transparency in the development of complex interventions in health care. The UCD process in this study can be used by researchers and designers as guidance for the development of mHealth interventions. However, taking time aspects into consideration, decisions have to be made about the thoroughness of executing individual phases.

## Appendix

Multimedia Appendix 1 Behavioral analysis of target behaviors and final intervention functions and behavior change techniques.

Multimedia Appendix 2 Content validity of the symptom monitoring module.

Multimedia Appendix 3 Demographic characteristics of participants during usability testing phases (phases 2 and 4).

Multimedia Appendix 4 Results field usability testing.

## Abbreviations

BCT: behavior change technique
BCW: behavior change wheel
COM-B: capability, opportunity, and motivation model of behavior
COPD: chronic obstructive pulmonary disease
eHealth: electronic health
HCP: health care provider
I-CVI: item-content validity index
MEDLINE: Medical Literature Analysis and Retrieval System Online
mHealth: mobile health
MRC: Medical Research Council
MVP: minimum viable product
RCT: randomized controlled trial
S-CVI: scale-content validity index
SUS: system usability scale
TDF: theoretical domains framework
UCD: user-centered design
