# Supplementary material for: User-Centered Design of a Mobile Health Intervention to Enhance Exacerbation-Related Self-Management in Patients With Chronic Obstructive Pulmonary Disease (Copilot): Mixed Methods Study
Source: J Med Internet Res. 2020 Jun 15;22(6):e15449. doi: 10.2196/15449 (PMC7324997; doi:10.2196/15449)
Supplement: Multimedia Appendix 1 [file jmir_v22i6e15449_app1.docx]

# APPENDIX 1: Behavioral analysis of target behaviors and final intervention functions and BCTs.

## Table 1. Target behavior: Self-monitoring of COPD symptoms and early detection of exacerbations.

| **COM-B** | **What needs to change to improve the target behavior** | **TDF** | **Intervention functions** | **BCTs** | **Demonstrated in MVP** |
| --- | --- | --- | --- | --- | --- |
| **Physical capability** | NA | NA | NA | NA | NA |
| **Psychological capability** | Have adequate knowledge on ‘normal’ pattern of COPD symptoms [1-3]. | Knowledge | Education | Information about health consequences; self-monitoring of behavior | (Daily) self-monitoring of normal symptom pattern; overview of normal symptoms in green zone action plan; overview of registered symptoms in calendar |
|  | Have the knowledge on what exacerbations are [1, 2, 4]. | Knowledge | Education | Information about health consequences | Information about COPD & exacerbations |
|  | Understand the importance of early detecting exacerbations [1]. | Knowledge | Education | Information about health consequences | Information about COPD & exacerbations |
|  | Have the knowledge (based on previous experiences) which symptoms are the best signals of deterioration [1]. | Knowledge | Education | Information about health consequences; self-monitoring of behavior | (Daily) self-monitoring of symptoms; overview of registered symptoms in calendar |
|  | Have the skills to explain his or her ‘normal’ symptom status and how this status can be recognized [5]. | Cognitive and interpersonal skills | Training | Self-monitoring of behavior | (Daily) self-monitoring of normal pattern of symptoms; overview of normal symptoms in green zone action plan |
|  | Have the skills to detect signals of symptom deterioration [1, 2, 4]. | Cognitive and interpersonal skills | Training | Self-monitoring of behavior; habit formation | (Daily) self-monitoring of symptoms; overview of registered symptoms in calendar; notification to fill out symptoms |
|  | Have the ability to use experiential knowledge to detect new exacerbations [1]. | Memory, attention and decision process | Training | Self-monitoring of behavior | (Daily) self-monitoring of symptoms; overview of registered symptoms in calendar |
| **Physical opportunity** | NA | NA | NA | NA | NA |
| **Social opportunity** | Have a competent HCP (e.g. competent in providing self-management support, providing clear guidance and tailored advice) [1, 3, 4, 6-8]. | Social influences | Enablement | Social support (practical) | HCP gives instruction about how to use the app, personalizes the action plan together with the patient and evaluates the app together with the patient |
|  | Have the opportunity to involve his or her social environment to support exacerbation detection [1, 5, 7, 9]. | Social influences | Enablement | Social support (unspecified) | Conversational interface (advice to consult social environment to discuss symptoms) |
| **Reflective motivation** | Trust own capacity to adequately detect exacerbations [1, 8, 9]. | Beliefs about capabilities | Education; persuasion; enablement | Self-monitoring of behavior; feedback on behavior; problem solving | (Daily) self-monitoring of symptoms; conversational interface (feedback on color zone selection); support for selecting color zone (grey zone) |
|  | Perceive that an exacerbation as a serious event [1, 2, 10]. | Beliefs about consequences | Education; persuasion | Information about health consequences; credible source | Information about COPD & exacerbations; a priori counselling by HCP on exacerbation-related self-management |
|  | Perceive that self-monitoring is important for own health [8]. | Beliefs about consequences | Education; persuasion | Information about health consequences; feedback on behavior; credible source | Information about COPD & exacerbations; conversational interface (positive feedback on performance of self-monitoring); a priori counselling by HCP on exacerbation-related self-management |
|  | Is motivated to self-monitor symptoms [1, 8, 11]. | Intentions | Education; persuasion; incentivisation | Information about health consequences; credible source; feedback on behavior; social reward | Information about COPD & exacerbations; a priori counselling by HCP on exacerbation-related self-management; conversational interface (positive feedback on performance of self-monitoring); overview of monitoring results in calendar |
| **Automatic motivation** | Have the habit of monitoring symptoms themselves [11, 12]. | Reinforcement | Training; incentivisation; environmental restructuring | Feedback on behavior; habit formation; social reward; prompts and cues | Conversational interface (positive feedback on performance of self-monitoring); notification to fill out symptoms |
|  | Perceive positive feelings regarding self-monitoring [8]. | Emotion | Persuasion; incentivisation | Feedback on behavior, social reward | Conversational interface (positive feedback on performance of self-monitoring); overview of monitoring results in calendar |

Abbreviations: COM-B, Capability, Opportunity, Motivation, Behavior; BCTs, behavior change techniques; TDF, Theoretical Domains Framework; NA, not applicable

## Table 2. Target behavior: Taking prompt individualized self-management actions (according to action plan).

| **COM-B** | **What needs to change to improve the target behavior** | **TDF** | **Intervention functions** | **BCTs** | **Demonstrated in MVP** |
| --- | --- | --- | --- | --- | --- |
| **Physical capability** | Have the skills and physical ability to perform actions (e.g. breathing techniques, using inhalation medication) [3, 10, 13]. | *Physical skills* | *Training* | *Demonstration of the behavior, Instruction on how to perform a behavior, behavioral practice/rehearsal* | *This is not implemented in the MVP. In a future version of the app a specific module with instruction and demonstration of these skills should be included to stimulate behavioral practice* |
| **Psychological capability** | Understand the importance of taking prompt actions [1-3, 10]. | Knowledge | Education | Information about health consequences | Information about COPD & exacerbations. |
|  | Have the knowledge on which actions can be performed how and at what time [3]. | Knowledge | Education | Information about health consequences | Information about COPD & exacerbations; overview of agreements in action plan; conversational interface (real-time advice for actions based on personalized action plan) |
|  | Have the skills to make adequate decisions regarding individualized actions [1, 4, 13]. | Cognitive and interpersonal skills | Training | Feedback on behavior | Conversational interface (feedback on performance of actions) |
|  | Let feelings of fear and anxiety not result in inadequate actions [1, 5, 9, 14]. | Memory, attention and decision process | Enablement | Action planning | Personalized action plan; conversational interface (real-time advice for actions based on personalized action plan) |
|  | Make adequate decision to take actions based on individualized action plan [13, 14]. | Behavioral regulation | Education; training, enablement | Prompt and cues; self-monitoring of behavior; action planning | Conversational interface (real-time advice for actions based on personalized action plan); registration of performed actions; personalized action plan |
| **Physical opportunity** | NA | NA | NA | NA | NA |
| **Social opportunity** | Have positive support in taking actions from social environment [1, 7, 9]. | Social influences | Enablement | Social support (unspecified) | Conversational interface (advice to consult social environment for help) |
| **Reflective motivation** | Have confidence in managing changes in symptoms [9]. | Beliefs about capabilities | Persuasion; enablement | Feedback on behavior; action planning | Conversational interface (real-time advice for actions based on personalized action plan; positive feedback on performance of actions); personalized action plan |
|  | Believe that he or she can influence exacerbations/perceive control on the disease [1, 3, 9, 14]. | Beliefs about capabilities | Education; persuasion | Information about health consequences; feedback on the behavior | Information about COPD & exacerbations; conversational interface (positive feedback on performance of actions); overview of symptoms and performed actions in calendar |
|  | Is motivated to take actions [1, 8]. | Intentions | Education; persuasion; incentivisation | Information about health consequences; credible source; feedback on behavior; social reward | Information about COPD & exacerbations; a priori counselling by HCP on exacerbation-related self-management; conversational interface (positive feedback on performance of actions); overview of performed actions in calendar |
| **Automatic motivation** | Have the habit of taking individualized actions based on action plan [15]. | Reinforcement | Training, incentivisation; environmental restructuring | Feedback on the behavior; social reward; habit formation; prompts and cues | Conversational interface (positive feedback on performance of actions; request to fill out performed actions; real-time advice for actions based on personalized action plan) |

Abbreviations: COM-B, Capability, Opportunity, Motivation, Behavior; BCTs, behavior change techniques; TDF, Theoretical Domains Framework; NA, not applicable; Italic: not implemented in MVP.

## Table 3. Target behavior: Prompt contact with a health care provider.

| **COM-B** | **What needs to change to improve the target behavior** | **TDF** | **Intervention functions** | **BCTs** | **Reflected in MVP** |
| --- | --- | --- | --- | --- | --- |
| **Physical capability** | NA | NA | NA | NA | NA |
| **Psychological capability** | Understand the importance of prompt health care contact [1-3, 10]. | Knowledge | Education | Information about health consequences | Information about COPD & exacerbations |
|  | Have the knowledge on when to consult a HCP [1-3]. | Knowledge | Education | Information about health consequences | Information about COPD & exacerbations; overview of agreements in action plan; conversational interface (real-time advice to contact a HCP based on personalized action plan) |
|  | Have the knowledge on which HCP should be consulted [8, 16]. | Knowledge | Education | Information about health consequences | Individualized agreements in action plan on which HCP can be consulted; conversational interface (real-time advice to contact a HCP based on action plan) |
|  | Make adequate decision to contact a HCP promptly based on individualized action plan [1, 13, 14]. | Behavioral regulation | Education; training, enablement | Prompt and cues; self-monitoring of behavior; action planning | Conversational interface (real-time advice to contact a HCP based on personalized action plan); registration of performed actions; personalized action plan |
| **Physical opportunity** | Have an accessible HCP [1, 7-9]. | *Environmental context and resources* | *Environmental restructuring* | *Restructuring the social environment* | *This is not implemented in the MVP. In a future version of the app, attention should be paid to the accessibility of HCPs* |
| **Social opportunity** | Have a competent HCP (e.g. competent in providing self-management support, providing clear guidance and tailored advice) [1, 3, 4, 6-8]. | Social influences | Enablement | Social support (practical) | HCP gives instruction about how to use the app, personalizes the action plan together with the patient and makes individualized agreements on which HCP can be consulted |
|  | Experience no threshold to contact a HCP due to collaborative relationship with HCP [1, 2, 8, 9]. | Social influences | Enablement | Social support (practical); action planning | HCP makes individualized agreements on which HCP can be consulted; personalized action plan |
|  | Have positive support from social environment in consulting a HCP promptly [1, 7, 9]. | Social influences | Enablement | Social support (unspecified) | Conversational interface (advice to consult social environment for help) |
| **Reflective motivation** | Believe that he or she could influence exacerbations/perceive control on the disease [1, 3, 9, 14]. | Beliefs about capabilities | Education; persuasion | Information about health consequences; feedback on behavior | Information about COPD & exacerbations; conversational interface (positive feedback on performance of actions); overview of symptoms and performed actions in calendar |
|  | Have confidence in consulting a HCP promptly [1]. | Beliefs about capabilities | Enablement; Persuasion | Action planning; feedback on behavior | Personalized action plan; conversational interface (positive feedback on contacting a HCP) |
|  | Believe that they know how to overcome barriers to consult a HCP [2-4, 10, 13, 14, 16, 17]. | *Beliefs about capabilities* | *Education; persuasion; enablement* | *Feedback on behavior; information about other’s approval; social support (emotional); action planning* | *This is not implemented in the MVP. In a future version of the app a specific step should be included to identify patient beliefs that form a threshold to contact a HCP and to provide feedback and support to overcome this threshold* |
|  | Express doubts and concerns that form a barrier for prompt health care contact (believe that overcoming barriers for health care contact is important) [1, 9, 16]. | *Beliefs about consequences* | *Education; persuasion* | *Information about health consequences; feedback on behavior; information about other’s approval* | *This is not implemented in the MVP. In a future version of the app a specific step should be included to identify patient beliefs that form a threshold to contact a HCP and to provide feedback and support to overcome this threshold* |
|  | Believe that medical treatment is needed [1, 16, 17]. | Beliefs about consequences | Education; persuasion | Information about health consequences; feedback on behavior | Information about COPD & exacerbations; conversational interface (real-time advice to contact a HCP based on action plan) |
|  | Motivated to consult a HCP promptly [1, 16]. | Intentions | Education; persuasion, incentivisation | Information about health consequences; feedback on behavior; credible source; social reward | Information about COPD & exacerbations; conversational interface (positive feedback on contacting a HCP); overview of performed actions in calendar; a priori counselling by HCP on exacerbation-related self-management |
| **Automatic motivation** | Have the habit of contacting a HCP if needed [15]. | Reinforcement | Training, incentivisation; environmental restructuring | Feedback on the behavior; social reward; habit formation, prompts and cues | Conversational interface (positive feedback on performed HCP contact; request to fill out performed HCP contact; real-time advice to contact a HCP based on personalized action plan) |

Abbreviations: COM-B, Capability, Opportunity, Motivation, Behavior; BCTs, behavior change techniques; TDF, Theoretical Domains Framework; NA, not applicable; Italic: not implemented in MVP.

## References

1. Korpershoek Y, Vervoort S, Nijssen L, et al. Factors influencing exacerbation-related self-management in patients with COPD: a qualitative study. Int J Chron Obstruct Pulmon Dis 2016 Nov 28; 11:2977-2990. PMID: 27932877.

2. Adams R, Chavannes N, Jones K, et al. Exacerbations of chronic obstructive pulmonary disease--a patients' perspective. Prim Care Respir J 2006 Apr; 15(2):102-109. PMID: 16701769.

3. Hernandez P, Balter M, Bourbeau J, et al. Living with chronic obstructive pulmonary disease: a survey of patients' knowledge and attitudes. Respir Med 2009 Jul; 103(7):1004-1012. PMID: 19269150.

4. Kessler R, Stahl E, Vogelmeier C, et al. Patient understanding, detection, and experience of COPD exacerbations: an observational, interview-based study. Chest 2006 Jul; 130(1):133-142. PMID: 16840393.

5. Harrison SL, Apps L, Singh SJ, et al. 'Consumed by breathing' - a critical interpretive meta-synthesis of the qualitative literature. Chronic Illn 2014 Mar; 10(1):31-49. PMID: 24227018.

6. Wortz K, Cade A, Menard JR, et al. A qualitative study of patients' goals and expectations for self-management of COPD. Prim Care Respir J 2012 Dec; 21(4):384-391. PMID: 23138844.

7. Cicutto L, Brooks D, Henderson K. Self-care issues from the perspective of individuals with chronic obstructive pulmonary disease. Patient Educ Couns 2004 Nov; 55(2):168-176. PMID: 15530751.

8. Korpershoek YJG, Vervoort SCJM, Trappenburg JCA, et al. Perceptions of patients with chronic obstructive pulmonary disease and their health care providers towards using mHealth for self-management of exacerbations: a qualitative study. BMC Health Serv Res 2018 Oct 4; 18(1):757-018-3545-4. PMID: 30286761.

9. Disler RT, Gallagher RD, Davidson PM. Factors influencing self-management in chronic obstructive pulmonary disease: an integrative review. Int J Nurs Stud 2012 Feb; 49(2):230-242. PMID: 22154095.

10. Barnes N, Calverley PM, Kaplan A, et al. Chronic obstructive pulmonary disease and exacerbations: patient insights from the global Hidden Depths of COPD survey. BMC Pulm Med 2013 Aug 23; 13:54-2466-13-54. PMID: 23971625.

11. Huniche L, Dinesen B, Nielsen C, et al. Patients' use of self-monitored readings for managing everyday life with COPD: a qualitative study. Telemed J E Health 2013 May; 19(5):396-402. PMID: 23531094.

12. Perski O, Blandford A, West R, et al. Conceptualising engagement with digital behaviour change interventions: a systematic review using principles from critical interpretive synthesis. Transl Behav Med 2017 Jun; 7(2):254-267. PMID: 27966189.

13. Trappenburg JC, Schaap D, Monninkhof EM, et al. How do COPD patients respond to exacerbations?. BMC Pulm Med 2011 Aug 19; 11:43-2466-11-43. PMID: 21854576.

14. Dowson CA, Town GI, Frampton C, et al. Psychopathology and illness beliefs influence COPD self-management. J Psychosom Res 2004 Mar; 56(3):333-340. PMID: 15046971.

15. Bischoff EW, Hamd DH, Sedeno M, et al. Effects of written action plan adherence on COPD exacerbation recovery. Thorax 2011 Jan; 66(1):26-31. PMID: 21037270.

16. Gruffydd-Jones K, Langley-Johnson C, Dyer C, et al. What are the needs of patients following discharge from hospital after an acute exacerbation of chronic obstructive pulmonary disease (COPD)?. Prim Care Respir J 2007 Dec; 16(6):363-368. PMID: 18038104.

17. Williams V, Hardinge M, Ryan S, et al. Patients' experience of identifying and managing exacerbations in COPD: a qualitative study. NPJ Prim Care Respir Med 2014 Sep 18; 24:14062. PMID: 25372181.
