# Supplementary material for: User-Centered Design of a Mobile Health Intervention to Enhance Exacerbation-Related Self-Management in Patients With Chronic Obstructive Pulmonary Disease (Copilot): Mixed Methods Study
Source: J Med Internet Res. 2020 Jun 15;22(6):e15449. doi: 10.2196/15449 (PMC7324997; doi:10.2196/15449)
Supplement: Multimedia Appendix 2 [file jmir_v22i6e15449_app2.docx]

# Appendix 2: Content validity of the symptom monitoring module

The aim of the symptom monitoring module was ‘to determine the individual COPD patients’ normal day-to-day variability in symptoms to be able to set the patients normal symptom pattern.’ An initial module was developed by two researchers (YK & JT) based on studies focusing on the incidence and course of exacerbations at a symptom level [1-3], studies evaluating COPD action plans [4-6] and the Global Initiative for Chronic Obstructive Lung Disease (GOLD) report [7]. The symptom monitoring module was developed and assessed on content validity by following a stepwise procedure (see Figure 1). The content validity of the module was evaluated by experts in the field of COPD (n=8) according to the Lynn method [8]. To assess the content validity, a survey was developed in the online survey service SurveyMonkey (SurveyMonkey Inc., San Mateo, CA, USA).

## Content validity rating

The aim of the symptom monitoring module was ‘*to determine the normal symptom pattern of a patient with COPD’.* Each symptom was rated on *relevance* and *linguistics* by answering 4 questions:

1) Is the symptom relevant for measuring the construct?

2) Is the question relevant to measure the specific symptom?

3) Are the answering options relevant?

4) Is the interpretation of both the question and answering options clear?

All questions on *relevance* (question 1-3) were rated on a 4-point Likert-scale (1= not relevant, 4=relevant). *Linguistics* was determined by whether interpretation was clear (‘yes’ or ‘no’). Feedback was asked in case of rating 1 or 2 or in case of unclear interpretation. Furthermore, participants were asked whether important symptoms were missing to measure the construct.

## Data analysis

All ratings on *relevance (*questions 1-3) were dichotomized into not relevant (0=rating 1 and 2) and relevant (1=rating 3 and 4). Then, the Item-Content Validity Index (I-CVI) for each question was calculated by summing all scores of one question and dividing this by the number of experts. A score above 0.78 was considered to be relevant. Second, the Scale-Content Validity Index (S-CVI) was calculated by summing all I-CVIs for each question and dividing this by the number of items. A score above 0.90 was considered to be excellent. To analyze *linguistics*, the frequency of ‘yes’ and ‘no’ ratings was determined. Linguistics was considered to be clear when at least 75% of the expert panel rated clearness of interpretation as a ‘yes’.

## Decision rules

Symptoms that were rated as both relevant and clear (all I-CVIs > 0.78 and clear interpretation ≥ 75% yes), without suggestions for adjustments, were included in the final module. When suggestions for adjustments were given, the symptom was included in the next round. Furthermore, symptoms with one or more questions rated as not relevant or unclear (I-CVI ≤ 0.78 or < 75% yes) were included in the next round. A symptom was excluded when the first question was considered as not relevant (I-CVI ≤ 0.78), unless there were serious reasons within the research team to ask feedback on that symptom again in the next round.

## Results

The final module was completed after 2 experts rounds (see Figure 1). The expert panel consisted of four researchers in the field of COPD (n=4), respiratory nurse specialists (n=2) a pulmonologist (n=1) and a respiratory nurse (n=1). The initial questionnaire of 11 symptoms was reduced to 10 symptoms after round 1. In total, eight items were included in round 2. Six of these items were adjusted based on feedback. The other two items were rated as not relevant, but the research team had doubts about this outcome. After the second round, eight symptoms were rated as relevant and clear (all I-CVIs > 0.78 and clear interpretation ≥ 75% yes) (see Figure 1). The relevance of the final symptom monitoring module consisting of eight symptoms, determined by question 1-3, was considered to be high with S-CVIs ≥ 0.93 (S-SCVI question 1= 0.93; S-CVI question 2 = 0.98 and S-CVI question 3 =0.94). The final selection of questions for the symptom monitoring module is shown in table 1.

**Generation of questions**

By two researchers based on:

- Individual studies
- International GOLD standard

**Face validity**

By one respiratory nurse specialist

**Content validity round 1**

By a survey with eight experts in the field of COPD

**Content validity round 2**

By a survey with five experts in the field of COPD

**Final selection of questions for the symptom monitoring module:**

8 symptoms: dyspnea, wheezing, night-time symptoms, coughing, sputum volume, sputum purulence, sputum color and fatigue

**Initial questionnaire of symptoms:** n=11

**No adjustments were made**

**Included in final module:** n=2

**Excluded:** insufficient relevance: n=1

**Second round:** unclear relevance or adjusted questions: n=8

**Included in final module:** n=6

**Excluded:** insufficient relevance: n=2

**Figure 1** Development and content validity assessment of the symptom monitoring module

**Table 1** Final selection of questions for the symptom monitoring module

| **Symptoms** | **Questions^a^** |
| --- | --- |
|  |  |
| 1. Dyspnea | When did you experience shortness of breath in the past 24 hours?  Choose the answer that suits you best:  *No shortness of breath*  *Shortness of breath during strenuous activity (like walking the stairs or rushing)*  *Shortness of breath during moderate activity (like walking or domestic work)*  *Shortness of breath during light activity (like washing or dressing)*  *Shortness of breath when resting (like talking or sitting still)* |
| 2. Wheezing | How often did you experience wheezing in the past 24 hours?  *Never / Occasionally / Often / All the time* |
| 3. Night-time symptoms | Last night, how often did you wake up due to shortness of breath?  *Never / Occasionally / Often / All the time* |
| 4. Coughing | How often did you cough in the past 24 hours?  *Never / Occasionally / Often / All the time* |
| 5. Sputum volume | How much mucus did you bring up when coughing in the past 24 hours?  *None / A Little / A lot / Very much* |
| 6. Sputum purulence | Did you experience thick or tough mucus in the past 24 hours?  *No / Yes* |
| 7. Sputum color | Which color did your mucus have in the past 24 hours?  *Transparant / Grey / Yellow / Green / Brown* |
| 8. Fatigue | How tired were you in the past 24 hours?  *Not at all tired / A bit tired / Very tired / Exhausted* |

^a^ Questions are translated from Dutch to English for this publication. Content validity was assessed for the Dutch questionnaire.

## References

1. Aaron SD, Donaldson GC, Whitmore GA, et al. Time course and pattern of COPD exacerbation onset. Thorax 2012 Mar; 67(3):238-243. PMID: 22008189.

2. Wilkinson TM, Donaldson GC, Hurst JR, et al. Early therapy improves outcomes of exacerbations of chronic obstructive pulmonary disease. Am J Respir Crit Care Med 2004 Jun 15; 169(12):1298-1303. PMID: 14990395.

3. Seemungal TA, Donaldson GC, Bhowmik A, et al. Time course and recovery of exacerbations in patients with chronic obstructive pulmonary disease. Am J Respir Crit Care Med 2000 May; 161(5):1608-1613. PMID: 10806163.

4. Trappenburg JC, Monninkhof EM, Bourbeau J, et al. Effect of an action plan with ongoing support by a case manager on exacerbation-related outcome in patients with COPD: a multicentre randomised controlled trial. Thorax 2011 Nov; 66(11):977-984. PMID: 21785156.

5. Bourbeau J, Julien M, Maltais F, et al. Reduction of hospital utilization in patients with chronic obstructive pulmonary disease: a disease-specific self-management intervention. Arch Intern Med 2003 Mar 10; 163(5):585-591. PMID: 12622605.

6. Lenferink A, Frith P, van der Valk P, et al. A self-management approach using self-initiated action plans for symptoms with ongoing nurse support in patients with Chronic Obstructive Pulmonary Disease (COPD) and comorbidities: the COPE-III study protocol. Contemp Clin Trials 2013 Sep; 36(1):81-89. PMID: 23770110.

7. Global Initiative for Chronic Obstructive Lung Disease (GOLD). Global Strategy for Prevention, Diagnosis and Management of COPD 2018; URL: https://goldcopd.org/wp-content/uploads/2017/11/GOLD-2018-v6.0-FINAL-revised-20-Nov_WMS.pdf. [accessed 2018-06-12].

8. Lynn MR. Determination and quantification of content validity. Nurs Res 1986 Nov-Dec; 35(6):382-385. PMID: 3640358.
