# Supplementary material for: User-Centered Design of a Mobile Health Intervention to Enhance Exacerbation-Related Self-Management in Patients With Chronic Obstructive Pulmonary Disease (Copilot): Mixed Methods Study
Source: J Med Internet Res. 2020 Jun 15;22(6):e15449. doi: 10.2196/15449 (PMC7324997; doi:10.2196/15449)
Supplement: Multimedia Appendix 3 [file jmir_v22i6e15449_app3.docx]

**Multimedia Appendix 3.** Demographic characteristics of participants during usability testing phases, (phases 2 and 4), COPD patients: n=24, professionals: n=9.

| Demographic characteristics | | | Phase 2A | Phase 2B | Phase 4 |
| --- | --- | --- | --- | --- | --- |
| **Characteristics of COPD^a^ patients** | | | | | |
|  | n | | 6 | 11 | 7 |
|  | **Sex, n=24 (100)** | | | | |
|  |  | Female | 3 (50) | 5 (45) | 5 (71) |
|  | Age (years), mean (SD) | | 62 (4.3) | 66 (7.8) | 58 (10.6) |
|  | **Ethnicity, n=13 (54)** | | | | |
|  |  | Dutch | 6 (100) | N/A^b^ | 7 (100) |
|  | **Living situation, n=13 (54)** | | | | |
|  |  | Alone | 2 (33) | N/A | 1 (14) |
|  | **Education level, n=13 (54)** | | | | |
|  |  | Low | 0 | N/A | 2 (29) |
|  |  | Medium | 4 (67) | N/A | 4 (57) |
|  |  | High | 2 (33) | N/A | 1 (14) |
|  | **Self-reported GOLD^c^ stage, n=24 (100)** | | | | |
|  |  | 1-2 | 0 | 4 (36) | 0 |
|  |  | 3-4 | 5 (83) | 7 (64) | 5 (71) |
|  |  | Unknown | 1 (17) | 0 | 2 (29) |
|  | **Average number of exacerbations per year, n=13 (54)** | | | | |
|  |  | ≤3 | 3 (50) | N/A | 4 (57) |
|  |  | >3 | 2 (33) | N/A | 0 |
|  |  | Unknown | 1 (17) | N/A | 3 (43) |
|  | **Mobile technology use, n=13 (54)** | | | | |
|  |  | Smartphone | 3 (50) | N/A | 4 (57) |
|  |  | Smartphone and tablet | 3 (50) | N/A | 3 (43) |
|  | **Mobile technology use for health purposes, n=13 (54**) | | | | |
|  |  | Yes | 3 (50) | N/A | 2 (29) |
| **Characteristics of health care providers, n=9 (100)** | | | | | |
|  | n | | 6 | N/A | 3 |
|  | **Sex, n=9 (100)** | | | | |
|  |  | Female | 5 (83) | N/A | 2 (67) |
|  | Age (years), mean (SD) | | 43 (12.3) | N/A | N/A |
|  | **Profession, n=9 (100)** | | | | |
|  |  | Primary care nurse | 1 (17) | N/A | N/A |
|  |  | General practitioner | N/A | N/A | 1 (33) |
|  |  | Respiratory nurse specialist (secondary care) | 1 (17) | N/A | 1 (33) |
|  |  | Respiratory nurse (secondary/tertiary care) | 2 (33) | N/A | 1 (33) |
|  |  | Clinical nurse (secondary care) | 1 (17) | N/A | N/A |
|  |  | Physician assistant (tertiary care) | 1 (17) | N/A | N/A |
|  | Work experience (years), mean (SD) | | 14 (9.4) | N/A | N/A |
|  | **Patient** **GOLD category most frequently cared for, n=6 (67)** | | | | |
|  |  | 1-2 | 1 (17) | N/A | N/A |
|  |  | 2-3 | 2 (33) | N/A | N/A |
|  |  | 3-4 | 3 (50) | N/A | N/A |
|  | **Use of mobile health in COPD care, n=6 (67)** | | | | |
|  |  | Yes | 2 (33) | N/A | N/A |
|  | **Use of written action plan in daily care, n=6 (67)** | | | | |
|  |  | Yes | 5 (83) | N/A | 1 (33) |

^a^COPD: chronic obstructive pulmonary disease.

^b^N/A: not applicable.

^c^GOLD: Global Initiative for Chronic Obstructive Lung Disease**.**
