# Supplementary material for: User-Centered Design of a Mobile Health Intervention to Enhance Exacerbation-Related Self-Management in Patients With Chronic Obstructive Pulmonary Disease (Copilot): Mixed Methods Study
Source: J Med Internet Res. 2020 Jun 15;22(6):e15449. doi: 10.2196/15449 (PMC7324997; doi:10.2196/15449)
Supplement: Multimedia Appendix 4 [file jmir_v22i6e15449_app4.docx]

# Appendix 4: Results field usability testing

**Table 1** Task success of COPD patients

| Task category | Task | Task succes^a^ | n |
| --- | --- | --- | --- |
| *Monitor symptoms and undertaken actions* | Fill out in the app that you feel well today, like you normally feel. | 1 | 4 |
|  |  | 0,5 | 1 |
|  | Imagine: You are feeling worse than the day before. Fill out how you feel in the app and check which actions you should undertake. | 1 | 3 |
|  |  | 0,5 | 1 |
|  | Imagine: You feel worse since two days, you experience more symptoms than you normally do ( increased dyspnea and more coughing). Fill this out in the app and view the actions you should undertake. | 1 | 1 |
|  |  | 0,5 | 1 |
|  |  | 0 | 1 |
|  | Imagine that you still feel less well and you would like to fill out which actions you have undertaken. Fill out the actions in the app and save these actions. | 1 | 5 |
|  |  | 0,5 | 1 |
|  |  | 0 | 1 |
|  | Turn of the daily notifications to fill out your symptoms by turning of the symptom diary. | 1 | 5 |
|  |  | 0,5 | 1 |
|  |  | 0 | 1 |
|  | Imagine: You have doubts about how you are feeling on a new day. Fill this out in the app and register your symptoms. | 1 | 1 |
|  |  | 0,5 | 1 |
|  |  | 0 | 2 |
|  | After completing registration in the grey zone, choose a color zone that corresponds with how you are feeling. | 1 | 2 |
|  |  | 0 | 1 |
| *View and adjust action plan* | Imagine that you need medical help immediately. Use the app as you would do in this case. | 1 | 6 |
|  |  | 0 | 1 |
|  | View the agreements you made with your HCP about the actions you should undertake when you feel well, like you normally do. | 1 | 5 |
|  | You would like to check the agreements you made with your HCP about the actions in the yellow zone. Look up these actions in the app. | 1 | 3 |
|  |  | 0,5 | 1 |
|  | Look up the agreements you made with your HCP about the actions you should undertake in the orange zone. | 1 | 2 |
|  |  | 0 | 1 |
|  | Look up the HCP you should contact if needed. | 1 | 7 |
|  | Imagine: You would like to adjust the symptoms in the green zone. Adjust these symptoms in the action plan. | 1 | 2 |
| *Review symptoms and actions (calendar)* | Look up in the app how you felt last week. | 1 | 5 |
|  |  | 0,5 | 2 |
|  | Imagine: You would like to show your physician how you felt yesterday and which actions you have undertaken at that time. Show this in the app. | 1 | 4 |
|  |  | 0,5 | 2 |
| *Read information (information module)* | You would like to know more about this app. Look up the information ‘about the app’. | 1 | 6 |
|  |  | 0,5 | 1 |
|  | You would like to know more about COPD and exacerbations. Look up this information in the app. | 1 | 4 |
|  |  | 0,5 | 1 |
|  |  | 0 | 2 |

^a^ performance of tasks: 1 point = successful; 0,5 point = partially successful; 0 points = unsuccessful.

**Table 2** Task success of HCPs

| Task category | Task | Task succes^a^ | n |
| --- | --- | --- | --- |
| *Personalize action plan* | Set up and personalize action plan. | 1 | 2 |
|  |  | 0,5 | 1 |
|  | Adjust action plan (green zone). | 1 | 2 |
|  |  | 0,5 | 1 |
|  | Adjust medication in action plan (yellow zone). | 1 | 2 |
|  | Add contact person to action plan. | 0 | 3 |
| *Review calendar* | Evaluate symptom monitoring period. | 1 | 2 |
| *Look up information* | Look up the information in the app. | 1 | 2 |

^a^ performance of tasks: 1 point = successful; 0,5 point = partially successful; 0 points = unsuccessful.

**Table 3** Observed user errors and problems in COPD patients

| User errors and problems | App location | Severity (0-4)^a^ | Freq (n) |
| --- | --- | --- | --- |
| 1. The saving button after symptom registration is overlooked/unclear. | Symptom monitoring | 4 | 5 |
| 2. Symptoms were unchecked without noticing. | Symptom monitoring | 4 | 1 |
| 3. Choosing the grey zone is not intuitive. | Choice for color zone | 4 | 3 |
| 4. The questions following in the grey zone appear too fast. | Conversation grey zone | 4 | 2 |
| 5. There are too many questions asked/unclear what users have to do. | Conversation grey zone | 4 | 2 |
| 6. The questions in the grey zone could not be read due to pop-up screen. | Conversation grey zone | 4 | 1 |
| 7. Symptoms in green zone could not be unchecked. | Action plan | 4 | 1 |
| 8. It is unclear that the action plan is an overview of symptoms due to similarities in lay-out with symptom registration. | Action plan - overview | 3 | 4 |
| 9. It is unclear that the ‘today’ button can be used to fill out symptoms. | Home screen | 3 | 1 |
| 10. It is unclear that the ‘today’ button can be used to register actions. | Home screen | 3 | 2 |
| 11. Changing contact details is not intuitive.^b^ | Contact details | 3 | 1 |
| 12. It is unclear that patients are in the yellow zone when experiencing one or more symptoms.^b^ | Action plan – color zone | 3 | 1 |
| 13. Actions cannot be found easily as users have to scroll down in the screen. | Action plan | 2 | 2 |
| 14. The color denotation of yellow and orange is not sufficiently distinctive. | Choice for color zone | 2 | 1 |
| 15. Symptom diary could not be found as scrolling down the screen is needed. | Symptom diary module | 2 | 3 |
| 16. In case of multiple symptom registration on one day, only the last registration can be found in the calendar. | Calendar | 2 | 1 |
| 17. Appointments for each color zone could not be found immediately. | Action plan | 2 | 1 |
| 18. It is unclear how medication in the action plan can be changed as users first have to go through the symptom list. | Change action plan | 2 | 1 |
| 19. Registered actions could not be found immediately. | Start screen | 2 | 2 |
| 20. It is unclear that symptom registration in the green zone means filling out ‘normal’ symptoms. | Symptom monitoring | 1 | 1 |
| 21. Information ‘about the app’ cannot be found immediately. | Start screen | 1 | 2 |
| 22. Expectations regarding the calendar function are unclear.^b^ | Calendar | 1 | 1 |
| 23. Information module ‘about the app’ does not include information about turning of the symptom diary. | Information ‘about the app’ | 1 | 1 |

^a^ 0 = no problem; 1 = cosmetic problem only; 2 = minor usability problem, 3 = major usability problem; 4 = usability catastrophe; ^b^ problems observed during free navigation in the app

**Table 4** Observed user errors and problems in HCPs

| User errors and problems | App location | Severity (0-4)^a^ | Freq (n) |
| --- | --- | --- | --- |
| 1. Green zone: The saving button after registration of symptoms is overlooked. | Personalize action plan | 4 | 2 |
| 2. Changing contact details is not intuitive.^b^ | Contact details | 4 | 3 |
| 3. Yellow zone: User clicks on symptoms. It is unclear that the yellow zone is an overview of symptoms that cannot be personalized. | Personalize action plan | 3 | 2 |
| 4. Change medication: When user forgets to save adjusted symptoms, the screen to change medication does not appear. | Change action plan | 3 | 1 |
| 5. User would like to distinguish short- and long-acting bronchodilators. | Fill out medication | 2 | 2 |
| 6. User fills out only a number to define medication dose. | Fill out medication | 2 | 2 |
| 7. Red button does not meet with current practice in primary care. | Action plan – red zone | 2 | 1 |
| 8. User would like to add a course of antibiotics and/or prednisolone to the orange zone. | Personalize action plan | 2 | 1 |
| 9. Onboarding text is not easy to read, user has to scroll down on the screen. | Onboarding app | 1 | 1 |
| 10. Onboarding text appears too fast. | Onboarding app | 1 | 1 |
| 11. Yellow zone: Unclear which medication should be added to the yellow zone as green zone includes also medication ‘if needed’. | Personalize action plan | 0 | 1 |

^a^ 0 = no problem; 1 = cosmetic problem only; 2 = minor usability problem, 3 = major usability problem; 4 = usability catastrophe; ^b^ problems observed during free navigation in the app

**Table 5** Patient and HCP perceptions towards using the app

| **Themes** | **Description** | **Quotes of COPD patients** |
| --- | --- | --- |
| **Feelings about the app** |  |  |
| *Feeling safe and confident* | A few patients mentioned that the app provided a certain feeling of safety and could increase their self-confidence. | *‘I would start using the app immediately. It’s the safety…and exactly knowing how you should act. A confident feeling that I can promptly take actions.’(P05)*  *‘I believe I would calmer…It gives me a more pleasant feeling, already. I would feel calmer when I would get the advice to raise the alarm. That increases my self-confidence.’(P3)* |
| *Being taken seriously* | Patients felt being taken seriously due to the conversation in the app that provides adequate feedback on registrations. | *‘I really like that! It feels like I am being taken seriously in a way. (P5)*  *‘I like it. It’s very personal. I have the idea that I am having a conversation with someone, that is more open and makes you feel better.’ (P7)* |
| *Feeling confident about using the app at home* | Most patients felt confident about using the app at home. One patient explained a need for assistance from relatives. | *‘I am 100% confident. If the app is installed on my phone, I can easily use it. No problem.’(P6)*  *‘I think I would have to learn how to use the app, but I could call my daughter, my wife or granddaughter for that.’ (P2)* |
| **Added value of the app** |  |  |
| *Increases awareness on wellbeing and taking adequate actions* | Most patients were convinced that the app would be helpful in detecting symptom deterioration and taking adequate actions. | *‘It is a useful tool to examine how you are really doing, you are not always aware of that.’ (P6)*  *‘I think it is an educational tool for myself. That I could learn to recognize when I need help and when I should take extra medication.’ (P1)*  *‘It would help me to go to the general practitioner on time. By filling out how I feel daily, I would realize early when it’s getting worse.’(P4)* |
| *Calendar provides insight into patient’s own situation* | All patients felt the calendar would provide insight into their own situation and would help them in conversations with HCPs. HCPs expressed that insights from the calendar could support making personalized agreements on self-management. | *‘I could use the calendar to show my doctor how I have been doing and how I have acted, because you won’t remember that. If I could use the app for that, that would be quit motivating (…) It is a reminder for myself as well, that I can check ‘How is my disease progressing?’. (P1)*  *‘I am positive about the colors that pop up in the calendar, so you can show the general practitioner ‘This is how it was doing.’ (P4)* |
| **Content of the app** |  |  |
| *Recognizable symptoms and advice* | Overall, patients were positive about the content of the app. Some patients trusted the app as they recognized the symptom color zones and the advices provided. | *‘The app seems reliable because you know what kind of answer you could expect yourself. And if it is right what you are thinking, and you are afraid to take actions, you could think ‘the app says it as well’. So it is actually more a confirmation you would ask…to feel sure.’(P3)* |
| *Additional measures and actions* | One patient expressed that registration of weight and physical activity would be nice. HCPs expressed a need for including self-treatment with prednisolone/AB. | *‘It would be nice if I could register my weight and if my sports program could be included in the app.’(P3)* |
| **Facilitators to use the app** |  |  |
| *Easy to use* | Most patients found the app easy the use and learned quickly how to work with the app. HCPs found the app easy to use as well, although it was somewhat unclear which parts of the action plan had to be personalized. | *‘It is all quite new, so you have to think about it…but it is quite compact and that makes it easy to use. I can’t say it is difficult, you are at the right place in only a few steps.’ (P1)*  *‘You would learn by using the app in daily practice.’(P3)*  *‘The first time you have to seek where everything is located. But, if you would use the app for a week, then it would be automatic. And using apps is not that easy for me in general.’(P4)* |
| *Personalized to own situation* | Patients were positive about the app being personalized to their situation. | *‘The app being personal, that is very positive. Like, it is ‘my green zone’, and not of someone else.’ (P06)* |
| *Agreement with HCP* | One patient expressed that agreement with a HCP to use the app would stimulate actual use. | *‘I would definitely use the app if I would agree with my health care provider on that.’(P06)* |
| **Barriers to use the app** |  |  |
| *Limited support during stable phase* | Two patients who rarely experienced exacerbations explained that the app would only have an added value if additional support is provided during the stable phase (green zone). | *‘I don’t experience that many symptoms at the moment. If I would suffer a lot, then I would use the app quite often. Now I would fill out ‘green’ all the time and then it would not be nice to use the app. So, therefore I would prefer to have more preventive components in the app. On the other hand, maybe I would use the app, because then I could examine how I am really feeling. I would like to try that.’(P06)* |
| *Lack of separate interface for HCPs* | One HCP expressed that a separate HCP interface to personalize and review the app would be helpful in daily practice. Another HCP however felt that using the app on the patients device would help to estimate if patients are able to use the app at home. | *NA* |
| *Further personalization of the app is needed* | Some patients mentioned that further personalization of the app by including personal comments would facilitate the use of the app. | *‘The app could be improved by making it even more personalized. So that the app could learn to know you better. (P01)*  *‘Maybe a diary could be included, so that you have free space to write down ‘I specifically experience this today.’ (P03)* |

**Textbox 1** Improvements for second version of the app

| **Problems that have to be solved for second version of the app**   - Saving symptoms in the app - Lay-out of action plan view (overview of symptoms and self-management actions) - Naming and explanation of buttons on home screen - Clarifying distinction between color zones - Clarifying which parts of the action plan should be individualized - Appearance of onboarding text in app - Selection of the grey zone and navigation in the grey zone - Changing contact details - Changing medication in action plan   **Functionalities that should be added to second version of the app**   - Further personalization of the content in the app and including free spaces for personal comments - Self-treatment (antibiotics and/or prednisolone) as self-management action in orange color zone - Differentiating short- and long acting bronchodilators - Assistance in case when patients are doubting about whether they should contact their HCP in case of symptom deterioration - Detailed feedback on importance of self-management actions in case of symptom deterioration - Including more preventive self-management actions in stable phase (green zone) - Including additional information on self-management actions in information module |
| --- |
